# Characterization of the HCN Interaction Partner TRIP8b/PEX5R in the Intracardiac Nervous System of TRIP8b-Deficient and Wild-Type Mice

**DOI:** 10.3390/ijms22094772

**Published:** 2021-04-30

**Authors:** Katharina Scherschel, Hanna Bräuninger, Andrea Mölders, Nadine Erlenhardt, Ehsan Amin, Christiane Jungen, Ulrike Pape, Diana Lindner, Dane M. Chetkovich, Nikolaj Klöcker, Christian Meyer

**Affiliations:** 1 Division of Cardiology, EVK Düsseldorf, cNEP, Cardiac Neuro- and Electrophysiology Research Consortium, Kirchfeldstrasse 40, 40217 Düsseldorf, Germany; katharina.scherschel@hhu.de; 2 Clinic for Cardiology, University Heart & Vascular Centre, University Hospital Hamburg-Eppendorf, Martinistrasse 52, 20246 Hamburg, Germany; h.braeuninger@uke.de (H.B.); c.jungen@uke.de (C.J.); ulrikepape1@gmail.com (U.P.); d.lindner@uke.de (D.L.); 3 DZHK (German Centre for Cardiovascular Research), Partner Site Hamburg/Kiel/Lübeck, 20246 Hamburg, Germany; 4 Institute of Neural and Sensory Physiology, Medical Faculty, Heinrich Heine University Düsseldorf, Universitätsstrasse 1, 40225 Düsseldorf, Germany; andrea.moelders@uni-duesseldorf.de (A.M.); nadine.erlenhardt@hhu.de (N.E.); ehsan.amin@hhu.de (E.A.); nikolaj.kloecker@uni-duesseldorf.de (N.K.); 5 Department of Cardiology, Leiden University Medical Center, 2333 ZA Leiden, The Netherlands; 6 Department of Neurology, Vanderbilt University Medical Center, Nashville, TN 37232, USA; dane.m.chetkovich@vumc.org

**Keywords:** autonomic nervous system, TRIP8b, HCN channels, cardiac electrophysiology

## Abstract

The tetratricopeptide repeat-containing Rab8b-interacting protein (TRIP8b/PEX5R) is an interaction partner and auxiliary subunit of hyperpolarization-activated cyclic nucleotide-gated (HCN) channels, which are key for rhythm generation in the brain and in the heart. Since TRIP8b is expressed in central neurons but not in cardiomyocytes, the TRIP8b-HCN interaction has been studied intensely in the brain, but is deemed irrelevant in the cardiac conduction system. Still, to date, TRIP8b has not been studied in the intrinsic cardiac nervous system (ICNS), a neuronal network located within epicardial fat pads. In vitro electrophysiological studies revealed that TRIP8b-deficient mouse hearts exhibit increased atrial refractory and atrioventricular nodal refractory periods, compared to hearts of wild-type littermates. Meanwhile, heart rate, sino-nodal recovery time, and ventricular refractory period did not differ between genotypes. Trip8b mRNA was detected in the ICNS by quantitative polymerase chain reaction. RNAscope in situ hybridization confirmed Trip8b localization in neuronal somata and nerve fibers. Additionally, we found a very low amount of mRNAs in the sinus node and atrioventricular node, most likely attributable to the delicate fibers innervating the conduction system. In contrast, TRIP8b protein was not detectable. Our data suggest that TRIP8b in the ICNS may play a role in the modulation of atrial electrophysiology beyond HCN-mediated sino-nodal control of the heart.

## 1. Introduction

In the brain and the heart, hyperpolarization-activated cyclic nucleotide-gated (HCN) channels are key for pacemaking [1,2]. The HCN interaction partner tetratricopeptide repeat-containing Rab8b-interacting protein (TRIP8b/PEX5R) is an auxiliary subunit, which affects the gating properties of HCN2 and HCN4, the main isoforms for spontaneous rhythm generation in cardiac pacemaker cells, by limiting cAMP-induced activation [3]. TRIP8b expression is cell- and tissue-restricted: in total, 11 splice forms are described in the central nervous system in selected neuronal [4] and glial [5] subpopulations and one in testis [6]. Previous studies have shown that TRIP8b is not expressed in the whole heart [6] or cardiomyocytes [3]. Still, sino-nodal pacemaker cells could be modulated by TRIP8b, as was recently shown with a minimal peptide that recapitulated the gating effect of full-length TRIP8b and was able to prevent cAMP regulation of HCN channels [7]. 

Cardiac rhythm and electrophysiology are modulated by the autonomic nervous system, which communicates signals from the brainstem to the intrinsic cardiac nervous system (ICNS) [8,9]. This complex network of different neurons and glial cells is located in atrial fat tissue and further relays signals via an extensive network of nerve fibers to the sinus node, atrioventricular (AV) node, and to the atrial and ventricular working myocardium. Even though cardiac neurons make up only a tiny amount of cells in the heart (approx. 1000 cells in the mouse [10], 43,000 in the human heart [11]), they modulate every heartbeat [8,12], and their molecular understanding is of high interest. The HCN current *I*_f_ has been recorded in the ICNS of guinea pigs [13,14], and action potential firing elicited by HCN2 was shown in other peripheral neurons [15]. HCN channels were shown to be present in parasympathetic [16], sympathetic [17], and sensory [15,18] ganglia of the autonomic nervous system. Thus, we hypothesized that the HCN channel auxiliary subunit TRIP8b is present in cardiac neurons of the ICNS and plays a role in the modulation of cardiac electrophysiology. 

Therefore, the goal of this study was to investigate TRIP8b in the murine ICNS using TRIP8b-deficient mice and wild-type littermate controls and perform in-depth expression analyses and in vitro electrophysiological studies. Our experiments reveal changes in atrial electrophysiology of TRIP8b-deficient mice, suggesting a role of TRIP8b in the ICNS beyond HCN-mediated sino-nodal control of the heart.

## 2. Results

### 2.1. Electrophysiological Recordings from Wild-Type and TRIP8b-Deficient Mouse Heart Preparations

**Figure 1:**
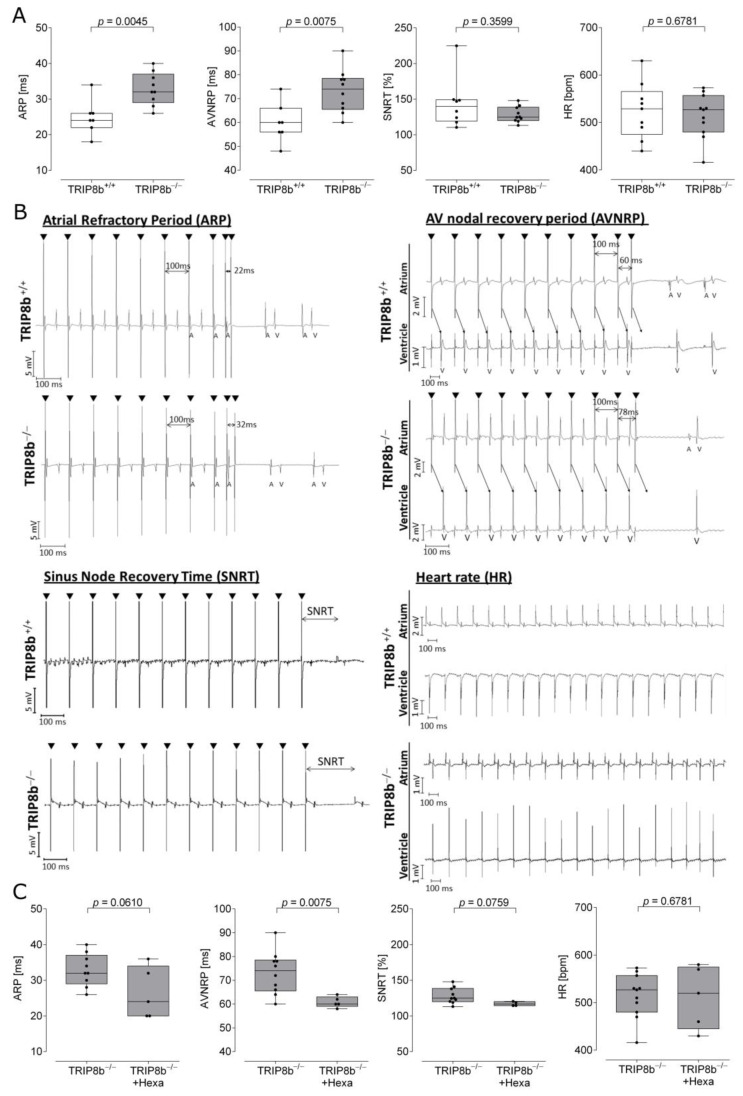
TRIP8b-deficient mice have altered atrial electrophysiology. (**A**) In vitro electrophysiological measurements from Langendorff-perfused hearts show an increase of atrial refractory period (ARP) and atrioventricular-nodal refractory period (AVNRP) in TRIP8b-deficient mice, without changes in sino-nodal activity (sino-nodal recovery time, SNRT) and heart rate (HR). (**B**) Representative tracings are shown for wild-type and TRIP8b-deficient mice. A, atrial activity; atrium electrophysiological tracings from the atrium; V, ventricular activity; ventricle electrophysiological tracings from the ventricle; black arrowheads mark atrial or ventricular stimulation. (**C**) Ganglionic blockade with 0.5 mM hexamethonium leads to a reduction of AVNRP in TRIP8b-deficient mice. Data are presented as box plots (minimum to maximum, *n* = 5–11 per genotype) and were compared using an unpaired *t*-test or Mann–Whitney, as appropriate.

Electrophysiological characterization of TRIP8b-deficient mouse hearts and wild-type littermate controls (exemplary genotyping in Supplementary Figure S1A) was performed employing programmed stimulation in vitro (Figure 1A). Representative tracings are presented in Figure 1B. ARP and AVNRP were increased in TRIP8b-deficient mice (ARP: 24.9 ± 1.8 ms wild type (*n* = 7) vs. 32.9 ± 1.5 ms knockout (*n* = 9), *p* = 0.0045 unpaired *t*-test; AVNRP: 60.0 ± 3.2 ms wild type (*n* = 7) vs. 73.2 ± 2.8 ms knockout (*n* = 10), *p* = 0.0075 unpaired *t*-test), indicating that conduction properties of atrial cardiomyocytes and AV nodal conduction is affected by the deletion of TRIP8b. In contrast, heart rate at the beginning of the protocol did not differ between the genotypes (heart rate: 524.4 ± 19.9 beats per minute (bpm) wild type (*n* = 9) vs. 514.5 ± 14.02 bpm knockout (*n* = 11), *p* = 0.6781 unpaired *t*-test). Additionally, neither SNRT nor rate-corrected SNRT (cSNRT) differed between the genotypes (SNRT%: 144.3 ± 12.6 wild type vs. 128.2 ± 3.5 knockout, *p* = 0.3599 Mann–Whitney test; cSNRT: 69.8 ± 19.2 wild type vs. 59.2 ± 8.8 knockout, *p* = 0.8868, Mann–Whitney test), indicating no changes affecting the sinus node. WBP and VRP remained unchanged (Supplementary Table S1), indicating no changes in ventricular level in TRIP8b-deficient mice. 

Next, we infused the nicotinic antagonist hexamethonium in hearts from TRIP8b-deficient mice to evaluate whether the blockade of ganglionic activity in the ICNS influences cardiac electrophysiological parameters. As described previously in wild-type mice [19], we detected a decrease of AVNRP (73.2 ± 2.8 ms without (*n* = 6) vs. 60.8 ± 1.0 ms with hexamethonium (*n* = 5), *p =* 0.0103, unpaired *t*-test), WBP (82.6 ± 2.2 ms without vs. 74.8 ± 1.0 ms with hexamethonium, *p* = 0.0330, unpaired *t*-test) and trends toward reduction of ARP, SNRT% and WBP (compare Figure 1C and Supplementary Table S1). This indicates that ganglionic activity is not diminished in TRIP8b-deficient mice.

AF was inducible in 3/18 attempts (16.7%) in hearts from wild-type animals, while 0/21 stimulations induced AF in TRIP8b-deficient animals (*p* = 0.051; Supplementary Figure S1B).

### 2.2. Morphological Analyses

Heart weight to body weight ratio did not differ between wild-type and TRIP8b-deficient mice (Supplementary Figure S1C), indicating that the hearts were neither hyper- nor hypotrophic. Accordingly, H&E staining (Supplementary Figure S1D) for morphological assessment showed no obvious structural changes or signs of remodeling in hearts of TRIP8b-deficient animals. Additionally, no indication of fibrosis was detected via picrosirius red staining (Supplementary Figure S1D).

### 2.3. Gene Expression Analysis of Trip8b

**Figure 2:**
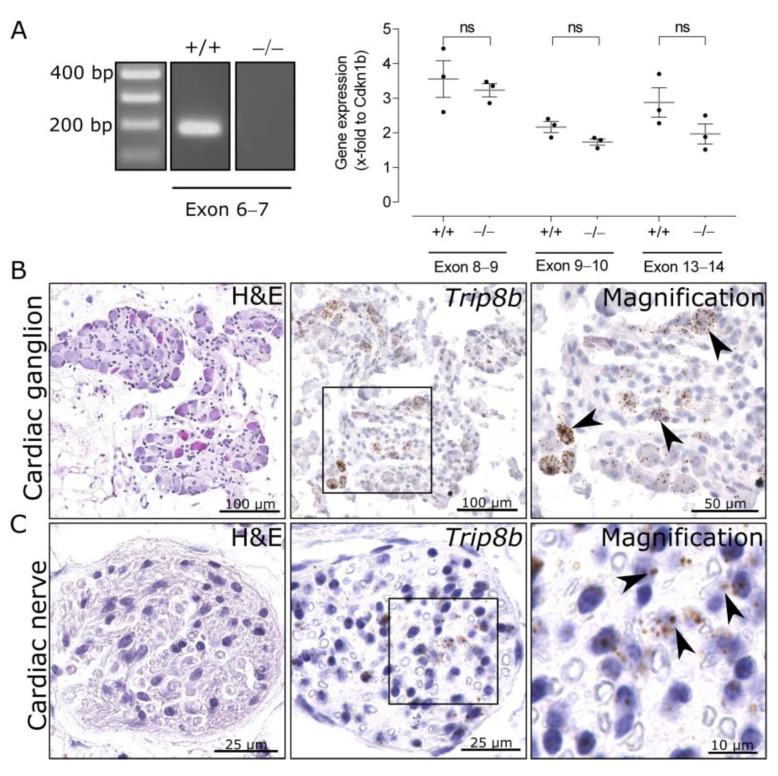
Trip8b mRNA is present in the cardiac nervous system. (**A**) Exon 6–7 can be amplified in cDNA of ganglia-containing atrial tissue of wild-type but not of TRIP8b-deficient mice (left panel). Quantitative PCR analyses show that exon 8–9, 9–10, and 13–14 of *Trip8b* are still detectable in knockout mice. Data (normalized to Cdkn1b) are presented as individual data points with SEM (*n* = 3, right panel) and were compared via one-way ANOVA followed by Sidaks’ multiple comparison test; ns, not significant. (**B**) *Trip8b* mRNA can be visualized with RNAscope in situ hybridization in neuronal cell bodies of cardiac ganglia. Black arrows in magnifications point to single neurons with *Trip8b* mRNAs. (**C**) *Trip8b* mRNA (black arrows) can be visualized with RNAscope in situ hybridization in cardiac nerves of wild-type mice.

Epicardial fat tissue from the atria, which contains cardiac nerves and neurons, was used for the generation of ICNS cDNA. A PCR with primers targeting exon 6/7, the deleted region in TRIP8b-deficient mice [4], was performed and resulted in a 200 bp band, indicating the presence of *Trip8b* mRNA in the ICNS in wild-type mice. As expected, there was no amplification product in knockout tissue (Figure 2A, left panel). Interestingly, quantitative PCR of ICNS cDNA detected exon 8–9, 9–10, and 13–14 of *Trip8b* in TRIP8b-deficient mice, even though there was a trend of a reduction of mRNA in knockout ICNS cDNA. CT values were exon 8–9: 32.5 ± 0.53 wild type vs. 32.84 ± 0.16 knockout; exon 9–10: 33.13 ± 0.44 wild type vs. 33.73 ± 0.05 knockout, exon 13–14: 32.6 ± 0.48 wild type vs. 33.43 ± 0.06 knockout (compare Figure 2A, right panel for relative expression normalized to *Cdkn1b*). This indicates the presence of partial *Trip8b* transcripts of later exons in knockout mice.

**Figure 3:**
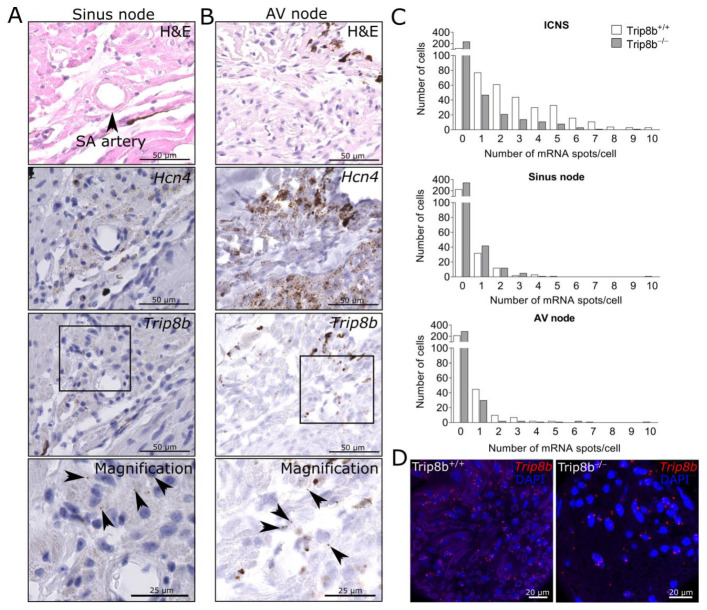
Trip8b mRNAs are detectable in the cardiac conduction system to a lower amount than in the intracardiac nervous system. (**A**) Sinus node and (**B**) atrioventricular node (AV node) were identified via hematoxylin and eosin (H&E) staining and *Hcn4* RNAscope in situ hybridization. Subsequent sections treated with a probe specific for *Trip8b* show solitary mRNA spots (black arrows) surrounding the sinus node artery and in the AV node. Nuclei are counterstained with hematoxylin in blue. (**C**) The histogram shows the distribution of *Trip8b* mRNA spots per cell in the intracardiac nervous system (ICNS, nerves, and ganglia), sinus node, and AV node of wild-type and TRIP8b-deficient mice. Overall, 279–404 cells were analyzed for each region of interest per genotype, *n* = 2–3 images/genotype. (**D**) *Trip8b* in situ hybridization (red) detects mRNA in wild-type mice but also, to a lower amount, in knockout mice.

To identify the location of *Trip8b*-expressing cells, mRNA was visualized via RNAscope in situ hybridizations of whole heart paraffin sections. As hypothesized, mRNA molecules were detectable in neuronal cell bodies within cardiac ganglia (Figure 2B) and cardiac nerves of the ICNS of wild-type animals (Figure 2C). The ICNS serves as a relay station for parasympathetic input from the brain to the sinus node and AV node, as well as the working myocardium [20]. Therefore, we analyzed the sinus node and AV node, identified by expression of *Hcn4*. We detected solitary *Trip8b* mRNAs in the sinus node surrounding the sinus node artery (Figure 3A) and the AV node (Figure 3B).

Quantification of *Trip8b* mRNA in ICNS, sinus node, and AV node was performed on images of fluorescent in situ hybridizations in both genotypes. Data are visualized as a histogram in Figure 3C. In the ICNS, we detected a mean of 2.18 ± 0.11 mRNA spots per cell vs. 0.73 ± 0.08 mRNA spots per cell in knockout (*p* < 0.0001, Mann–Whitney test; nerve: *n* = 3 images/genotype, ganglia: *n* = 3 images/wild type, *n* = 1 image/knockout). In sinus node, 0.32 ± 0.06 mRNA spots/cell were detected in wild type vs. 0.21 ± 0.03 mRNA spots/cell in knockout (*p* = 0.6972, Mann–Whitney test; *n* = 2 images/wild type, *n* = 3 images/knockout). In the AV node, 0.43 ± 0.06 mRNA spots/cell were present in wild type vs. 0.19 ± 0.04 mRNA spots/cell in knockout (*p* = 0.5859, Mann–Whitney test, *n* = 3 images/genotype). Between 279 and 404 cells were analyzed per region of interest and per genotype. The presence of *Trip8b* mRNAs in the ICNS of TRIP8b-deficient mice is presented as exemplary in Figure 3D. This is in line with the results from quantitative PCR, confirming the presence of partial transcripts. The quantification also confirms the visual assessment that more *Trip8b* is present in the ICNS than in the conduction system in wild-type mice (*p* < 0.0001, Kruskal–Wallis tests between ICNS, sinus node, and AV node). In the ICNS, 70.1% of wild-type cells presented at least one *Trip8b* mRNA spot (vs. 31.1% in knockout), in sinus node 18.4% in wild type (vs. 14.9% in knockout) and in the AV node 24.3% in wild type (vs. 11.7% in knockout). The high percentage of *Trip8b* expressing cells in the ICNS, compared to the conduction system, indicates that this is the origin of the phenotype in TRIP8b-deficient mice.

### 2.4. Protein Expression Analysis of TRIP8b

**Figure 4:**
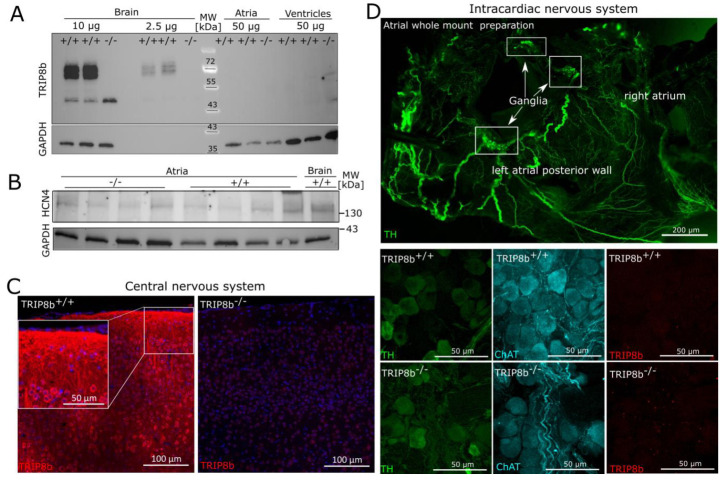
TRIP8b protein is not detectable in the atrial lysates and the cardiac autonomic nervous system. (**A**) Western blot analysis of brain tissue as positive control detects specific bands for TRIP8b (NBP2-38840, Novusbio) already at 2.5 µg total protein. For the heart, 50 µg atrial or ventricular lysate did not show any specific bands, while HCN4 (**B**) is detectable in both genotypes. (**C**) Immunohistochemistry for TRIP8b (APR-070, Alomone Labs) on paraffin sections was established in the central nervous system, more specifically, the cerebral cortex. Neurons positive for TRIP8b are detectable in the wild-type animals but not cortex of TRIP8b-deficient animals. (**D**) To increase the sensitivity of detection, atrial whole-mount preparations (upper panel shows exemplary staining with αTH ab152, Merck Millipore) were stained and ganglia cut out for confocal microscopy (bottom panel with αTH ab76442, Abcam). No specific signal was obtained for TRIP8b (APR-070, Alomone Labs), and no differences were detectable between the genotypes.

Protein expression of TRIP8b was analyzed via immunoblotting. As expected, a double band of approx. 72 kDa was detectable in wild-type brain lysates [4] with 10 µg and 2.5 µg of the total protein lacking in the knockout brain (Figure 4A, Supplementary Figure S2). In contrast, no specific signals were obtained in atrial lysates, including fat tissue and cardiac neurons, as well as in ventricular lysates (Figure 4A), while Western blot analysis for HCN4 detected a weak band at approx. 130 kDa in wild-type and TRIP8b-deficient animals (Figure 4B). This was verified using a different TRIP8b antibody (Alomone Labs, Jerusalem, Israel) with the same result.

**Figure 5:**
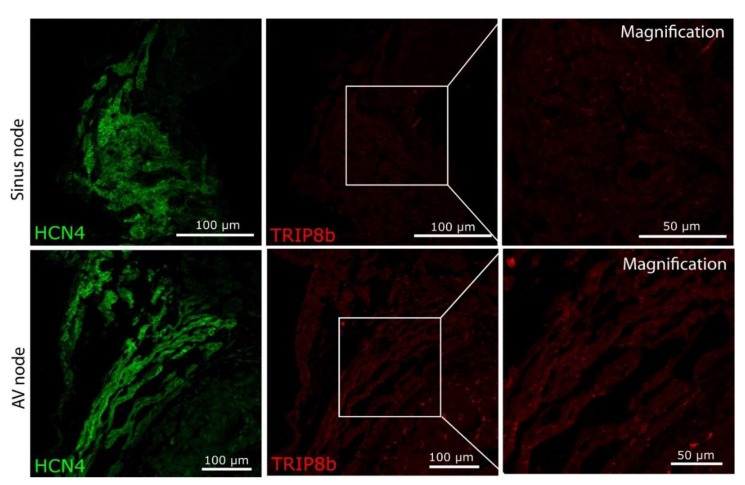
TRIP8b protein is not detectable in the cardiac conduction system in wild-type mice. Sinus node (upper panel) and atrioventricular node (AV node, bottom panel) were identified by anatomical landmarks and HCN4 staining (green). No staining for TRIP8b (red, APR-070, Alomone Labs) was detectable beyond the background.

Therefore, we established TRIP8b immunohistological staining on paraffin sections in the wild-type brain, which showed distinct expression patterns in the cerebral cortex, as expected (Figure 4C). Still, no specific staining was obtained in cardiac ganglia on paraffin sections; therefore, we performed whole-mount staining of atrial preparations (Figure 4D, upper panel) and dissected complete cardiac ganglia under a fluorescence binocular for subsequent confocal microscopy to increase the sensitivity of detection. Still, neither did we detect any TRIP8b staining beyond the background, nor did the staining differ between cardiac ganglia of wild-type and TRIP8b-deficient mice (Figure 4D, bottom panel). In line with this, no specific signal was obtained in the working myocardium, as well as sinus node (Figure 5, upper panel) and AV node of both genotypes (Figure 5, lower panel).

### 2.5. HCN Channel Expression Analysis in Cardiac Ganglia

**Figure 6:**
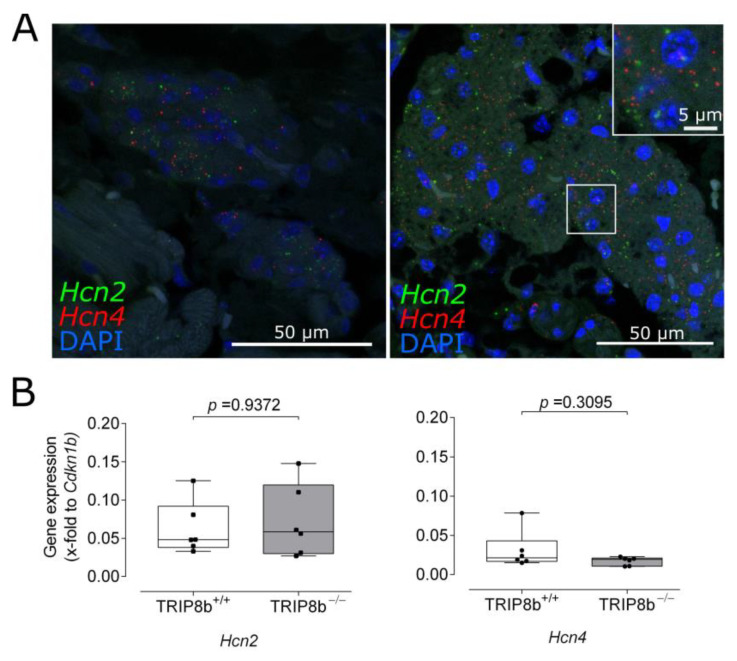
HCN channel expression in intracardiac ganglia. (**A**) In situ hybridization of two exemplary wild-type ganglia for *Hcn2* (green) and *Hcn4* (red). Both mRNAs are present within the ganglia. Boxed area is magnified in the inlay. (**B**) Gene expression analysis of *Hcn2* and *Hcn4* in TRIP8b-deficient mice and wild-type littermates. Data are presented as normalized gene expression to *Cdkn1b* using the formula 2^−ΔCt^ (box plots, minimum to maximum, *n* = 6 per genotype) and were compared using Mann–Whitney test.

Since the main known function of TRIP8b to date is the modulation of HCN channels by affecting gating properties of HCN2 and HCN4 by limiting cAMP-induced activation [3], we studied the expression of Hcn2 and Hcn4 in intracardiac ganglia. In situ hybridization on paraffin sections showed that both mRNAs are present in ganglia of wild-type mice (Figure 6A). Gene expression of Hcn2 and Hcn4 normalized to Cdkn1b, measured by quantitative PCR in ICNS lysates, did not differ between the genotypes (Figure 6B; Hcn2: 0.06 ± 0.01 wild type vs. 0.07 ± 0.02 knockout; *n* = 6 per genotype; *p* = 0.9371; data compared with Mann–Whitney test; Hcn4: 0.03 ± 0.01 wild type vs. 0.02 ± 0.002 knockout; *n* = 6 per genotype; *p* = 0.3095; data compared with Mann–Whitney test). This indicates that the electrophysiological phenotype of TRIP8b-deficient mice is most likely not attributable to changes in HCN expression levels.

## 3. Discussion

The ICNS is important for the modulation of cardiac electrophysiology, and the HCN current *I*_f_ has been recorded in cardiac neurons of guinea pigs [13,14]. TRIP8b is an interaction partner for HCN channels [6] and an auxiliary subunit that, upon assembly with HCN2 and HCN4, limits their activation by cAMP [3,21,22]. Here, we studied the expression and function of the HCN channel auxiliary subunit TRIP8b in the ICNS. Our main results are (1) TRIP8b-deficient mouse hearts show changes in atrial electrophysiology but not in sino-nodal function. (2) *Trip8b* mRNA is reliably detected in neuronal somata and fibers of the ICNS and, sparsely, in the sinus node and the AV node, most likely in nerve fibers within this region. Taken together, our experiments suggest a role of TRIP8b in the modulation of atrial electrophysiology that is beyond HCN-mediated sino-nodal control of the heart.

Modulation of cardiac electrophysiology has already been shown for other auxiliary subunits of HCN channels, such as vesicle-associated membrane protein (VAMP)-associated protein B (VAPB/ERG30) [23]. Still, VAPB is expressed in the working myocardium, which is not the case for TRIP8b, according to the state of literature [6] and our own findings. In good agreement, no structural abnormalities such as myocardial alterations or remodeling were detectable in hearts from TRIP8b-deficient mice, which could have been a potential explanation for electrophysiological changes [24]. Electrophysiological characterization of TRIP8b-deficient mice in our study demonstrated increased ARP and AVNRP, but no changes in sino-nodal conduction, heart rate, or other common electrophysiological parameters connected to changes in the cholinergic system [19], compared to wild-type littermates. It is surprising that we observed an effect in AV-nodal but not in sino-nodal conduction, but it was shown before that HCN channel inhibition influences the AV node much more than the sinus node, even though a higher amount of HCN4 channels are expressed in the latter [25]. Neurons of the ICNS have been shown to modify atrial and ventricular refractory periods directly [19,26]. A modulation of membrane resting potential and firing patterns by HCN channels has been described in other peripheral neurons, e.g., for HCN2 [15], which we show to be present in the ICNS as well. It could be speculated that the loss of TRIP8b leads to changes in neuronal firing, thereby affecting ARP via the ICNS without changing sino-nodal activity. The Langendorff model was shown to be well suited for studies of the intracardiac nervous system [19,27,28]. Indeed, the ICNS is embedded in autonomic reflex circuits, which can only be studied in vivo, thus limiting our conclusions.

We detected *Trip8b* mRNA in the ICNS via quantitative PCR and in situ hybridization. Santoro et al. have shown that *Trip8b* mRNA was not present in the heart [6], but these analyses were performed via Northern blotting, most likely of whole hearts, and did therefore contain only a little or no parts of the ICNS, which is located in epicardial fat tissue surrounding the atria. We were able to identify neuronal somata and nerve fibers of the ICNS as the origin of *Trip8b* mRNA. Additionally, a very low amount of mRNAs was found in the sinus node and AV node. Since both sinus and AV nodes are highly innervated by the autonomic nervous system [20], it is very likely that the observed *Trip8b* signal instead derives from single fine nerve fibers in this region. Of note is that, while exons 6–7 were lacking, transcripts from exons 8–9, 9–10, and 13–14 of *Trip8b* were detectable in knockout ICNS, indicating that splicing across the deletion took place. This also explains why RNAscope detects *Trip8b* also in TRIP8b-deficient animals since the employed technique typically uses approx. 20 different probes covering a region of one kb on the RNA molecule [29]. While these transcripts might lead to the generation of truncated protein forms, this was negated by Lewis et al. using antibodies against the N- and C-terminus of TRIP8b [4]. 

We were able to detect TRIP8b protein in the brain by Western blotting and immunohistochemistry, both in alignment with the literature [4,6]. As cardiac neurons account for only approximately 1000 cells in the mouse heart [10], expression of TRIP8b in the ICNS might be insufficient for detection by Western blotting. Still, even though immunohistological staining of TRIP8b in the brain gave reproducible results, TRIP8b was neither detectable in intracardiac ganglia nor in nerves, sinus node, AV node, or atrial or ventricular working myocardium, suggesting that the antibodies might be insufficient. It could also be speculated that TRIP8b protein is downregulated in the healthy heart and upregulated under (pathophysiological) circumstances or as needed, as was shown for the expression—and subsequent functional activity—of HCN channels in autonomic ganglia in diabetic mice [30]. However, evaluating whether *Trip8b* mRNA in the ICNS is detectable in a disease setting or in other autonomic ganglia was beyond the scope of this study.

Parasympathetic activation is highly relevant for atrial electrophysiology; in general, it shortens ARP and thereby increases the risk for atrial arrhythmias [31,32]. The higher ARP observed in TRIP8b-deficient animals might therefore present a potential anti-arrhythmic mechanism. Still, since no protein was found in the ICNS of wild-type mice that could explain this phenotype in the knockout. As cardiac autonomic control (sympathetic and parasympathetic) is conveyed from the brainstem [8], it could also be hypothesized that extracardiac reflex circuits in form of changes in neurotransmitter levels or differentially expressed proteins may be responsible for changes in atrial electrophysiology of TRIP8b-deficient mice. Susceptibility to AF did not differ between the genotypes, but this might be due to the fact that healthy mice are not prone to arrhythmias and thus the number of successful inductions was low in our study. A potential influence of TRIP8b on arrhythmia susceptibility should be tested in mice with an increased risk [33]. 

In conclusion, our study suggests a role of TRIP8b in the modulation of atrial electrophysiology beyond sino-nodal control.

## 4. Materials and Methods

### 4.1. TRIP8b-Deficient Mice

TRIP8b^−/−^ mice (C57Bl/6J) originally derived from Lewis et al. were graciously provided from Prof. Dr. Budde, Westfälische Wilhelms-Universität Münster and genotyped as described [4]. All experiments were performed in accordance with the Guide for the Care and Use of Laboratory Animals published by the US National Research Council Committee (8th edition, updated 2011) and approved by the regional regulatory authorities.

### 4.2. Langendorff Perfusion

Langendorff experiments and electrophysiological characterization using programmed stimulation were performed with male TRIP8b^−/−^ mice and respective wild-type littermates (14–22 weeks), as described previously [19]. Mice were anesthetized by inhalation of isoflurane (Forane, Abbott Laboratories, Hannover, Germany 3–5%). Adequate anesthesia was verified by loss of pedal withdrawal reflex before mice were euthanized via cervical dislocation. Hearts were excised and prepared for perfusion with modified Krebs-Henseleit solution: 119 mM NaCl, 25 mM NaHCO_3_, 4.6 mM KCl, 1.2 mM KH_2_PO_4_, 1.1 mM MgSO_4_, 2.5 mM CaCl_2_, 8.3 mM glucose, and 2 mM Na pyruvate; pH 7.4, 95% O2/5% CO_2_. A 2F octapolar electrophysiology catheter (CIB’ER Mouse, NuMED Inc., Cross Roads, TX, USA) was inserted into the right ventricle for electrical stimulation with a cycle length of 100 ms for the initial 20-min equilibration period. 0.5 mM hexamethonium (Sigma-Aldrich, St. Louis, MO, USA), a nicotinic antagonist, was added to the perfusion buffer before equilibration to block the activity of the intracardiac ganglia. 

### 4.3. In Vitro Electrophysiological Study

Programmed stimulation was applied using a designated digital stimulus generator (STG4002, Multi-Channel Systems, Reutlingen, Germany) at twice the atrial or ventricular pacing threshold to obtain standard electrophysiological parameters [19,34].

Atrial (ARP) and ventricular refractory periods (VRP) were determined as the longest extrastimulus cycle length with an absent atrial or ventricular response (12 stimuli; S1S1: 100 ms; one short-coupled extrastimulus with a 2 ms stepwise S1S2 reduction). Atrio-ventricular-nodal refractory period (AVNRP) was determined as longest extrastimulus cycle length with loss of AV-nodal conduction (12 stimuli; S1S1: 100 ms; one short-coupled extrastimulus with a 2 ms stepwise S1S2 reduction), Wenckebach periodicity (WBP) as the longest S1S1 cycle length with loss of 1:1 AV-nodal conduction (8 stimuli; S1S1: 100 ms; 2 ms stepwise reduction). Sinus node recovery time (SNRT) was determined as the maximum return cycle length after 10 s of fixed-rate pacing at an S1S1 cycle length of 120 ms, 100 ms, and 80 ms. Rate-corrected SNRT (cSNRT) was calculated by subtracting the sinus cycle length from SNRT. For induction of atrial arrhythmias, burst stimulation was performed with twice the atrial pacing threshold according to Schrickel et al. (5 s at S1S1: 50–10 ms, 10 ms stepwise reduction) [35,36]. Atrial fibrillation (AF) was defined as rapid atrial electrogram for more than 1 s. 

### 4.4. Histology and Immunohistochemistry

Langendorff-perfused hearts were formalin-fixed for 24–48 h at RT, dehydrated, paraffin-embedded, and cut into 4 µm sections for all subsequent experiments. Sections were deparaffinized and rehydrated. Hematoxylin–eosin (H&E) was used for the detection of cardiac ganglia. Picrosirius red staining for visualization of fibrosis was performed using reagents from Polysciences Inc. (Warrington, PA, USA) according to the manufacturer’s instructions. Immunohistological staining was performed, as described in detail previously [19,37]. Briefly, heat-induced antigen retrieval was performed with citrate buffer (pH 6) in a pressure cooker followed by treatment with 0.25% Sudan black/70% ethanol for 30 min for quenching of autofluorescence. Sections were permeabilized (0.2% Triton X-100/ tris-buffered saline (TBS) for 10 min) and blocked (3% bovine serum albumin (BSA)/TBS for 1 h at RT) before primary antibody (Supplementary Table S2) incubation overnight at 4 °C in 1% BSA/TBS. After three TBS washes for 10 min each, secondary antibodies (Supplementary Table S3) were incubated for 2 h at RT in TBS. Slides were mounted in DAPI Fluoromount-G (SouthernBiotech, Birmingham, AL, USA). Brains were embedded in 5% agarose/PBS and sagitally cut at 30 µm using a vibratome.

### 4.5. Staining of Murine Atria and Vibratome Sections

Staining of intracardiac ganglia from atrial whole-mount preparations was adapted from previous protocols [19]. Even though cardiac ganglia are parasympathetic, they express tyrosine hydroxylase in a subset of neurons [19,38] which was used for identification due to the strong staining. Ganglia-containing fat pads were dissected from Langendorff-perfused and formalin-fixed hearts using fine forceps. Lung, esophagus, and trachea were removed and specimens stored in phosphate-buffered saline (PBS) at 4 °C. Brains were formalin-fixed for 48 h at room temperature, embedded in 5% agarose/PBS, and cut into 30 µm sagittal sections using a vibratome.

For immunohistological staining, incubation steps were performed in 96-well plates. Specimens were bleached in Dent’s bleach (MeOH:DMSO:H_2_O_2_ 4:1:1) [39] 2–12 h at 4 °C, rehydrated in a descending methanol series, and subsequently permeabilized by incubation 2 × 1 h in 1% Triton X-100 in PBS at RT. To decrease autofluorescence, Sudan black (0.25% in 70% ethanol) staining was performed for 2 h at RT. Atrial preparations were blocked overnight at 4 °C (5% BSA/PBS 0.1% Triton X-100). Primary antibodies (Supplementary Table S1) were incubated in a blocking buffer for 96 h at 4 °C. Specimens were washed 3 × 1 h in 0.1% Triton X-100/PBS. Incubation of secondary antibodies was performed in 0.1% Triton X-100/PBS for 48 h at 4 °C. Afterward, samples were washed for 6 × 30 min in 0.1% Triton X-100/PBS. Vibratome sections were mounted in DAPI Fluoromount, while atria were stored in PBS at 4 °C. 

### 4.6. RNAscope In Situ Hybridization

RNAscope in situ hybridization assay [29] was carried out for detection of *Trip8b/Pex5l*, *Hcn2,* and *Hcn4* mRNA on paraffin sections using RNAscope 2.5 HD detection kit (#322310; Advanced Cell Diagnostics (ACD), Newark, CA, USA) for chromogenic staining and RNAscope Multiplex Fluorescent v2 reagent kit (#323100, ACD, Newark, CA, USA) for fluorescent staining following the manufacturer’s instructions. In brief, sections were deparaffinized by incubation at 60 °C for 1 h in xylene replacement (HS200-5, Sigma-Aldrich St. Louis, MO, USA) and 100% ethanol, followed by 10 min hydrogen peroxide incubation to quench internal peroxidase activity and 10 min target retrieval at 95 °C. In addition, protease plus treatment at 40 °C for 30 min was performed to permeabilize tissue sections. The probe (RNAscope Mm-Pex5l #551531; Mm-Hcn4 #421271; Mm-Hcn2-C2 #427001-C2, ACD) was hybridized for 2 h at 40 °C, followed by RNAscope amplification steps. In the case of chromogenic staining 3,3′-diaminobenzidine and hematoxylin were used for visualization of staining and cell nucleus, respectively. Slides stained with the fluorescent RNAscope kit were mounted in DAPI Fluoromount-G for nucleus staining.

### 4.7. Microscopy

Confocal images were generated using a Leica TCS SP5 confocal microscope (Leica Microsystems GmbH, Wetzlar, Germany) with ×10 numerical aperture (NA) = 0.3 HC PL Fluotar, ×20 NA = 0.7 HC PL APO CS Imm/Corr oil, and ×40 NA = 1.3 HCX PL APO CS objectives. Three-dimensional images were collected over the full range of the signal, and a maximum projection image was created using the Leica LAS AF software. A Keyence digital microscope was used to image chromogenic sections (plan apo ×10 NA = 0.45 and plan apo ×60 NA = 1.40 oil objectives). The atrial whole mount was imaged using an SMZ25 stereomicroscope (Nikon, Tokyo, Japan).

### 4.8. mRNA Quantification

For mRNA quantification, the QuPath software, version 0.2.3 [40] was used on fluorescent RNAscope maximum z projection confocal images comprised of approx. five focal planes with a stack size of 0.5 µm adapted from Merritt et al. [41]. The regions of interest (ROI: ICNS [nerves and ganglia], sinus node, and AV node) were marked with the freehand tool to quantify the number of cells (nuclei, based on DAPI staining) and the number of spots per cell was computated by the software. As reported by Merritt et al., nucleus detection was not reliable in all cases; therefore, nuclei bigger than double standard deviation were excluded from the analysis. Due to differences in tissue composition, settings used for the detection of nuclei were set for each ROI differently, while detection of RNAscope signals was identical. The following settings were used for detection of nuclei: ‘requested pixel size’: 0.5; ‘background radius’: 8.0 (ICNS), 10 (sinus node), 8.0 (AV node); ‘median radius’: 0.0; ‘sigma’: 1.5; ‘minimum area’: 10.0; ‘maximum area’: 400.0; ‘threshold’: 25.0 (ICNS), 10.0 (sinus node) 10.0 (AV node); ‘split by shape’: true ‘cell expansion’: 5.0 (ICNS), 10.0 (sinus node), 5.0 (AV node); ‘include cell nucleus’: true; ‘smooth boundaries’: true; ‘make measurements’: true. RNAscope spot detection was performed with the following settings: ‘detection [Channel]’: 35.0; ‘Smooth before detection: true; ‘split by intensity’: true; ‘split by shape’: true; ‘expected spot Size’: 1.0; ‘min spot size’: 0.2; ‘max spot size’: 1.0; ‘include clusters’: true.

### 4.9. Western Blot Analysis

To study TRIP8b protein expression, Western blot analysis of cardiac tissue was performed with the brain as positive control [19]. Hearts were dissected as described above and atria, as well as ventricles, shock frozen in liquid nitrogen. Frozen tissues were ground using mortar and pestle. Powdered tissue was lysed in RIPA buffer (50 mM Tris base pH 8.0, 150 mM sodium chloride, 0.5% sodium deoxycholate, 0.1% sodium dodecyl sulfate, 1% Triton X-100, 1 mM dithiothreitol, plus protease inhibitors (Complete Mini, Roche Roche Life Science, Basel, Switzerland) for 1 h at ice and centrifuged for 30 min at 15.000 g, 4 °C. Protein concentrations were quantified using the Pierce BCA Protein Assay Kit (ThermoFisher Scientific, Waltham, MA, USA) according to the manufacturer’s instructions. 

Proteins were separated under denaturing conditions by sodium dodecyl sulfate-polyacrylamide gels (SDS–PAGE) using 10% acrylamide gels and transferred to nitrocellulose membranes via semidry blotting using the Trans-Blot Turbo Transfer System (Bio-Rad, Hercules, CA, USA). The membranes were blocked in 5% skim milk powder in TBS and incubated overnight at 4 °C with primary antibody (Supplementary Table S1). After three washing steps with TBS/0.5% Tween, a secondary antibody (Supplementary Table S2) was incubated for one hour at room temperature. The Fusion Solo S gel documentation system (VRW International, Radnor, PA, USA) was used to detect reactive protein bands with enhanced chemiluminescence (Super Signal West Femto, Thermo Fisher Scientific, Waltham, MA, USA).

### 4.10. Gene Expression Analyses

Tissue samples were snap frozen and lysed with QIAzol lysis reagent in a TissueLyser II (Qiagen, Hilden, Germany). RNA was extracted using the RNeasy mini kit (Qiagen, Hilden, Germany) following the manufacturer’s protocol, as described previously [19]. DNAse I treatment for 1 h was performed to avoid DNA contamination. RNA concentration was measured with the help of Nanodrop 2000c (ThermoFisher Scientific, Waltham, MA, USA), and RNA was stored at −80 °C until later usage. For reverse RNA transcription using the high capacity cDNAse kit (ThermoFisher Scientific, Waltham, MA, USA) 250 ng of total RNA was used. Generated cDNA was diluted to a working concentration of 2.5 ng/µL. For gene expression analyses real-time PCR was performed using 4 µL cDNA template, 5 µL gene expression master mix (ThermoFisher Scientific, Waltham, MA, USA), and 0.5 µL gene expression assay (Life Technologies) in a QuantStudio 7 Flex, Applied Biosystems (ThermoFisher Scientific, Waltham, MA, USA). Gene expression assays, containing forward and reverse primers and FAM-labelled probe, are listed in Supplementary Table S4. Measurements were performed in duplicates. Gene expression was compared by normalizing the gene expression to the endogenous control *Cdkn1b* using the formula 2^−ΔCt^.

### 4.11. Statistical Analyses

All values are described in mean ± standard error of the mean (SEM) if not stated otherwise. Data were tested for normality using the D’Agostino–Pearson omnibus test. Student’s *t*-test was used for comparisons of normally distributed data. Nonparametric tests were chosen for differences across groups with not normally distributed data. *p* values < 0.05 were considered statistically significant. Statistical analysis was performed using GraphPad Prism 6.07 (GraphPad Software, San Diego, CA, USA).

## Data Availability

Not applicable.

## Supplementary Materials

The following is available online at https://www.mdpi.com/article/10.3390/ijms22094772/s1.
