# Supplementary material for: Characterization of the HCN Interaction Partner TRIP8b/PEX5R in the Intracardiac Nervous System of TRIP8b-Deficient and Wild-Type Mice
Source: Int J Mol Sci. 2021 Apr 30;22(9):4772. doi: 10.3390/ijms22094772 (PMC8125662; doi:10.3390/ijms22094772)
Supplement: Supplementary file 1 [file ijms-22-04772-s001.zip › ijms-1187626-supplementary.pdf]

Supplementary Material

Characterization of the HCN Interaction Partner TRIP8b/PEX5R in the Intracardiac Nervous System of TRIP8b-Deficient and Wild-Type Mice

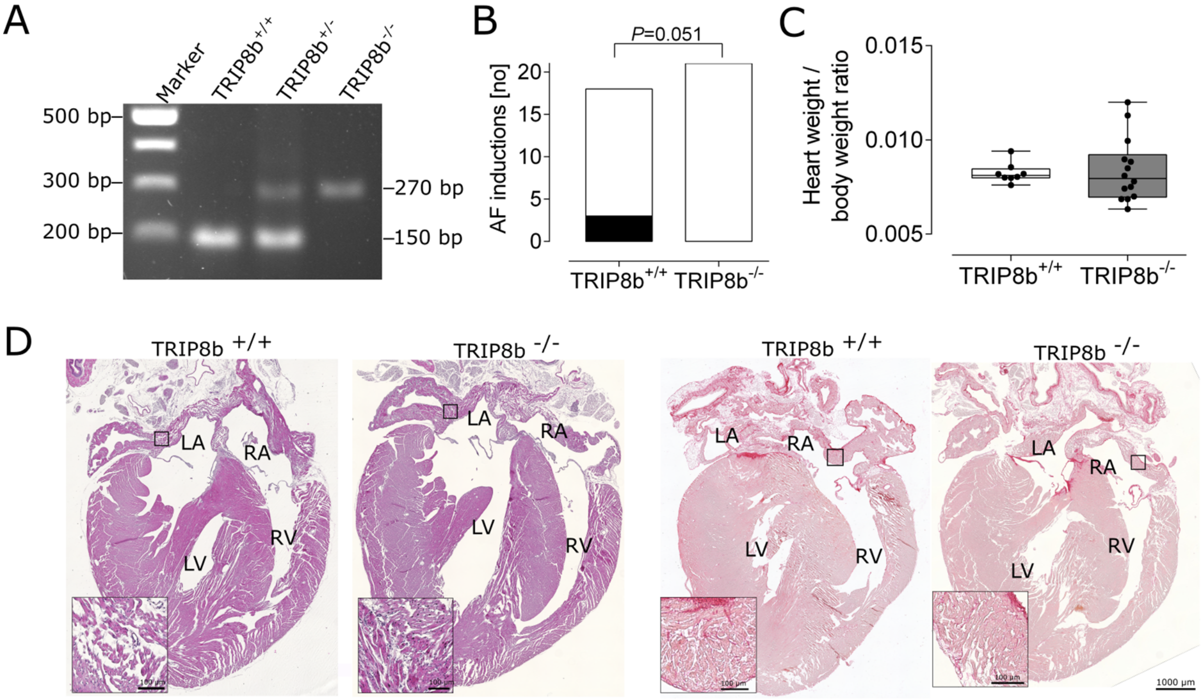

Figure S1. (**A**) Genotyping PCR for TRIP8b-deficient mice according to Lewis et al. [4]. See Supplementary Table S5 for primer sequences. (**B**) Atrial fibrillation (AF) was induced in 3 (black part of the graph) of 18 attempts (white part of the graph) in wild type mice, and in 0/21 attempts in TRIP8b-deficient mice (n = 8 wild type, n = 9 TRIP8b-deficient animals, chi square test). (**C**) Heart weight to body weight ratio does not differ between wild-type and TRIP8b-deficient animals. (**D**) Left panel H&E staining for general morphology and right panel picrosirius red staining for detection of fibrosis do not reveal any structural abnormalities in TRIP8b-deficient mice.

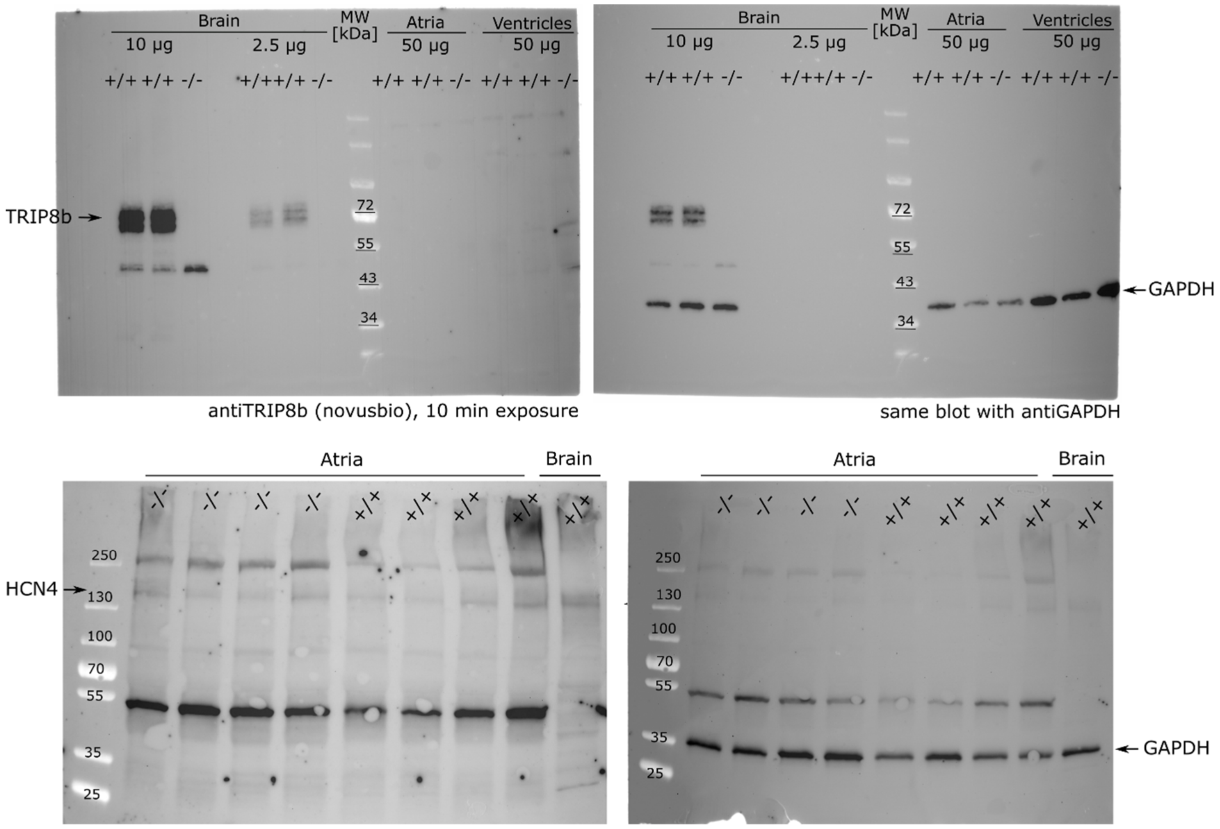

Figure S2. Uncropped western blots for TRIP8b (anti TRIP8b, NBP2-38840, novusbio, upper panel) and HCN4 (APC-052, alomone labs, lower panel) from Figure 4A. Blots are presented with the corresponding GAPDH signal on the same membrane.

Table S1. In vitro electrophysiological characterization of TRIP8b-deficient mice with and without Hexamethonium.

**Parameter**	**TRIP8b$^{+/+}$**	**TRIP8b$^{-/-}$**	***p* Value ($^{+/+}$ vs. $^{-/-}$)**	**TRIP8b$^{-/-}$ + Hexa**	***P* value ($^{-/-}$ vs. $^{-/-}$ + Hexa)**
ARP	24.9 ± 1.8	32.9 ± 1.5	0.0045	26.4 ± 3.3	0.0610
AVNRP	60.0 ± 3.2	73.2 ± 2.8	0.0075	60.8 ± 1.0	0.0103
HR	524.4 ± 19.9	514.5 ± 14.02	0.6781	512.0 ± 29.6	0.6781
SNRT%	144.3 ± 12.6	128.2 ± 3.5	0. 3599	117.1 ± 1.6	0.0759
SNRTc	69.8 ± 19.2	59.2 ± 8.8	0.8868	34.3 ± 4.34	0.1059
WBP	76.3 ± 3.7	82.6 ± 2.2	0.1423	74.8 ± 1.0	0.0339
VRP	28.2 ± 2.0	29.64 ± 1.5	0.5663	28.4 ± 1.9	0.6314

n = 5–10 per genotype. Data compared with Mann-Whitney or unpaired *t*-test as appropriate. ARP atrial refractory period; AVNRP atrio-ventricular nodal refractory period; HR heart rate; SNRT sino-nodal recovery time; SNRTc heart-rate corrected SNRT; WBP Wenckebach periodicity; VRP ventricular refractory period.

Table S2. Primary antibodies.

Antigen	Species	Company	Ordering Number	Dilution IHC	Dilution WB	Application
TH	rabbit	Merck-Millipore	ab152	1:1000	N/A	WM over-view
TH	chicken	Abcam	ab76442	1:500	N/A	WM
ChAT	goat	Merck-Millipore	ab144P	1:500	N/A	WM
HCN4	mouse	Neuromab	75-150	1:100	N/A	IHC
HCN4	rabbit	Alomone labs	APC-052	N/A	1:250	WB
TRIP8b	rabbit	Alomone labs	APR-070	1:4000	1:1000	IHC, WB (*)
TRIP8b	rabbit	Novusbio	NBP2-38840	N/A	1:1000	WB
TRIP8b	mouse	Neuromab	75-244	1:1000	N/A	IHC (*)
GAPDH-HRP	rabbit	Cell Signaling	14c10	N/A	1:2000	WB

IHC immunohistochemistry; WB western blot; WM whole mount staining. (*) used to reproduce results generated with the other TRIP8b antibodies, data not shown.

Table S3. Secondary antibodies.

Antigen	Species	Label	Company	Ordering Number	Dilution
Rabbit IgG	Donkey	488	Life technologies	A21206	1:500
Mouse IgG1	Goat	568	Life technologies	A21124	1:500
Mouse IgG	Donkey	568	Life technologies	A10037	1:500
Chicken IgY	Donkey	647	Merck-Millipore	AP194SA6	1:500
Goat IgG	Donkey	568	Life technologies	A11057	1:500
Goat IgG	Donkey	647	Life technologies	A21447	1:500
Rabbit IgG	Goat	HRP	Vector laboratories	PI-1000-1	1:10,000

HRP Horse radish peroxidase.

Table S4. *TaqMan* gene expression probes.

TaqMan Probe	Gene Name	Ordering Number
Cdkn1b	Cyclin-dependent kinase inhibitor 1b	Mm00438167_g1
Hcn2	Hyperpolarization-activated cyclic nucleotide-gated channel 2	Mm00468538_m1
Hcn4	Hyperpolarization-activated cyclic nucleotide-gated channel 4	Mm01176086_m1
Pex5l/Trip8b Exon 8/9	Tetratricopeptide repeat-containing Rab8b-interacting protein	Mm00458083_m1
Pex5l/Trip8b Exon 9/10	Tetratricopeptide repeat-containing Rab8b-interacting protein	Mm01284162_m1
Pex5l/Trip8b Exon 13/14	Tetratricopeptide repeat-containing Rab8b-interacting protein	Mm00458088_m1

Table S5. Primer sequences.

Name	Sequence 5′ 3′	Utilization
Sense (TSKC5′)	GCCCAATTGATGCATTTACTTTGG	Genotyping
Anti-Sense (1.1b3′)	TGTGCCTATGTCTGCCTTCCCAG	Genotyping
Anti-Sense (TSKB3′)	CTGGACACAAACTAGAGTCACGG	Genotyping
Sense (Trip8b Exon 6)	CTTGACCTGAGTGAACCCGT	PCR
Anti-Sense (Trip8b Exon 7)	CCAGTTGTGTTTGAATGTCTAAATC	PCR
